# Evolution of Laboratory Diagnostics for Cryptococcosis and Missing Links to Optimize Diagnosis and Outcomes in Resource-Constrained Settings

**DOI:** 10.1093/ofid/ofae487

**Published:** 2024-08-27

**Authors:** Richard Kwizera, Tadeo K Kiiza, Andrew Akampurira, Sarah Kimuda, Timothy Mugabi, David B Meya

**Affiliations:** Infectious Diseases Institute, College of Health Sciences, Makerere University, Kampala, Uganda; Department of Medical Microbiology, School of Biomedical Sciences, College of Health Sciences, Makerere University, Kampala, Uganda; Infectious Diseases Institute, College of Health Sciences, Makerere University, Kampala, Uganda; Infectious Diseases Institute, College of Health Sciences, Makerere University, Kampala, Uganda; Department of Medical Microbiology, School of Biomedical Sciences, College of Health Sciences, Makerere University, Kampala, Uganda; Infectious Diseases Institute, College of Health Sciences, Makerere University, Kampala, Uganda; Infectious Diseases Institute, College of Health Sciences, Makerere University, Kampala, Uganda; Infectious Diseases Institute, College of Health Sciences, Makerere University, Kampala, Uganda; Department of Medicine, School of Medicine, College of Health Sciences, Makerere University, Kampala, Uganda

**Keywords:** cryptococcal meningitis, cryptococcosis, fungal diagnostics, laboratory diagnostics, resource-constrained setting

## Abstract

Cryptococcal meningitis is one of the leading causes of death in sub-Saharan Africa among patients with advanced HIV disease. Early diagnosis is crucial in improving treatment outcomes. Despite advances and the availability of modern and point-of-care diagnostics for cryptococcosis, gaps still exist in resource-constrained settings, leading to unfavorable treatment outcomes. Here, we review the current outstanding issues or missing links that need to be filled to optimize the diagnosis of cryptococcosis in resource-constrained settings to improve treatment outcomes. We highlight the evolution of cryptococcosis diagnostics; the roles of early fungicidal activity, cryptococcal antigen titers, antifungal susceptibility testing, and therapeutic drug monitoring; and the missing links to optimize diagnosis and outcomes, including practical recommendations.

## Teaser

The major missing links to optimize treatment outcomes among patients with cryptococcal meningitis involve troubleshooting the limitations of fungal culture, addressing delayed diagnosis, and developing more versatile diagnostics, as well as improving access to the cryptococcal antigen lateral flow assay, antifungal susceptibility testing, and therapeutic drug monitoring. Improved human resource capacity is still key. Procurement issues, cost of tests, and competing diseases and health systems of these settings also hinder access to essential diagnostics for cryptococcal meningitis.

*Cryptococcosis* refers to a spectrum of infections (mostly meningitis) caused by *Cryptococcus neoformans* in patients who are immunocompromised [1]. *Cryptococcus gattii* is known to cause disease (mostly pneumonia) in individuals who are immunocompetent. However, *C gattii* may cause meningitis. Infection in humans occurs when basidiospores are inhaled into the lungs. The spores are deposited into the alveoli and germinate to establish a dormant infection. Once a host becomes immunocompromised, dissemination from the lungs via blood throughout the body occurs. While there is a perception that this is a neurotropic yeast, on autopsy yeast is widely disseminated throughout the body. Once disseminated, antigens can be detected in blood, and viable cryptococcus cells can be recovered by culturing the cerebrospinal fluid (CSF) of affected individuals [2]. Cryptococcal meningitis occurs when the membranes that cover the brain and spinal cord, known as *meninges*, are infected.

HIV with a CD4 cell count <200 cells/μL is a major risk factor [3]. Cryptococcal antigenemia is present in about 4% to 8% of patients with advanced HIV disease [4, 5]. Cryptococcal meningitis is one of the leading causes of death in sub-Saharan Africa among individuals with advanced HIV disease and significant morbidity and mortality. It is responsible for 19% of AIDS-related mortality globally [5]. Early diagnosis is crucial in improving treatment outcomes.

Timely diagnosis of cryptococcal meningitis reduces the mortality rate in patients with advanced HIV disease and morbidity associated with the disease [6]. Diagnosis of cryptococcal meningitis currently relies on recovery of the fungus in CSF. Fungal culture is the historical gold standard. Detection of cryptococcal antigen (CrAg) in CSF by point-of-care tests has recently improved the diagnosis of cryptococcal meningitis. CrAgs can be detected in other body fluids, such as whole blood, serum, plasma, urine, and saliva, with deferring diagnostic performances [7–9].

However, despite advances and the availability of point-of-care diagnostics for cryptococcosis, gaps still exist in diagnostics in resource-constrained settings (RCS), leading to unfavorable treatment outcomes. We sought to review the current outstanding issues or missing links that need to be filled to optimize the diagnosis of cryptococcosis to improve treatment outcomes in RCS.

## EVOLUTION OF CRYPTOCOCCOSIS DIAGNOSTICS

Diagnostics for cryptococcosis has evolved over time, from complex laboratory-based tests to simple tests that can be used in field conditions and at the bedside or point of care.

### Fungal CSF Culture

**Figure 1.**
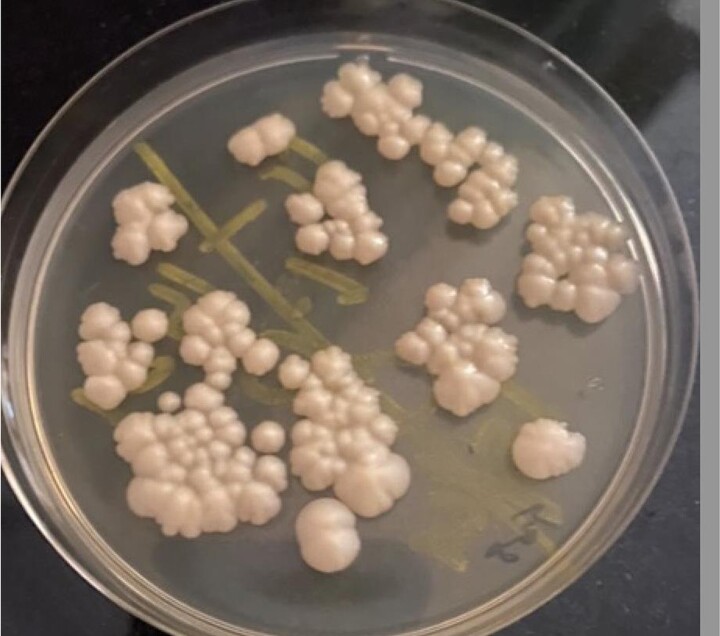
Fungal culture. *Cryptococcus neoformans* isolates from cerebrospinal fluid on Sabouraud dextrose agar with chloramphenicol.

Fungal culture of CSF is historically considered the gold standard for diagnosis of cryptococcal meningitis. Quantitative cryptococcal cultures can be used to monitor fungal burden and disease progression. Culture has a turnaround time of 7 to 14 days. Any fungal growth before the maximum culture time can be reported and treatment started for the patients. When fungal burden is low, culture can give false-negative results [9]; thus, culture is no longer considered the gold standard. Culture can differentiate between cryptococcal meningitis relapse and paradoxical cryptococcal immune reconstitution inflammatory syndrome (IRIS). However, culture needs laboratory infrastructure. Culture is the most available fungal test in RCS [10], but facilities need to have the capacity to do lumbar punctures (Figure 1).

There are different types of media used in the isolation of *Cryptococcus* species. The most common ones include Sabouraud dextrose agar, CHROMagar, bird seed agar, and urea agar test. Sabouraud dextrose agar is a nonselective medium used for the isolation of pathogenic and nonpathogenic fungi from clinical and nonclinical specimens. CHROMagar is a selective chromogenic culture medium used in the qualitative direct detection, differentiation, and presumptive identification of yeasts [11]. Bird seed agar is used for selective isolation and differentiation of *C neoformans* from other *Cryptococcus* species and yeasts [12]. The urease agar test is used for the rapid presumptive identification of *C neoformans* [13]. Other differential media include L-canavanine glycine bromothymol blue agar, which has the ability to differentiate most *C gattii* isolates from *C neoformans* [14].

### Other Preliminary CSF Parameters

As culture has a long turnaround time, preliminary analyses are done on CSF to give a presumptive diagnosis for cryptococcal meningitis. These include CSF color, clarity, viscosity, proteins, glucose, white cell count, and red blood cell count. All these parameters are nonspecific and must be measured as soon as possible after a lumbar puncture.

### CSF Color

Normal CSF is expected to be colorless. The analysis is usually macroscopic by simple visual inspection of a sample held in front of a white piece of paper. Changes in the color of CSF are not diagnostic but may indicate additional substances in the sample. The term *xanthochromia* is used to describe abnormal CSF pigmentation, with a slight pink, yellow, or orange discoloration [15]. This indicates the presence of pigmented compounds such as oxyhemoglobin, bilirubin, and methemoglobin, which are usually derived from the breakdown of red blood cells. It can be suggestive of subarachnoid bleeding. Green CSF may also be seen with bilirubin or infection [16]. Patients with cryptococcal meningitis usually have colorless CSF.

### CSF Appearance

Cloudy or turbid CSF may indicate the presence of white or red blood cells, microbes, or an increase in protein levels. Patients with cryptococcal meningitis usually have clear CSF, but a cloudy appearance is more common in bacterial meningitis, tuberculous meningitis, or a coinfection of cryptococcal meningitis and bacterial meningitis or tuberculous meningitis.

### CSF Viscosity

Normal CSF will have the same consistency as water. CSF that is “thicker” may indicate meningitis. Patients with cryptococcal meningitis occasionally have thicker CSF, but this is not conclusive. The median CSF viscosity relative to water has been shown to be 1.1, and CSF viscosity in baseline and serial CSF samples correlated with fungal burden [17].

### CSF Proteins

CSF proteins are analyzed by measuring the optical density with a spectrophotometer and then compared with a standard curve of known protein concentrations [18]. Decreases in CSF protein are not generally considered significant. CSF protein levels exhibit high variability in HIV-associated cryptococcal meningitis, from being normal to markedly elevated. Approximately one-third of the patients have a baseline CSF protein ≥100 mg/dL [19].

### CSF Glucose

CSF glucose levels may be lower when cells that are not normally present in the central nervous system use it up [20]. While not diagnostic, low glucose levels are seen in cryptococcal meningitis. However, reduced CSF glucose has been reported to be more prevalent in tuberculous meningitis [21].

### CSF White Blood Cell Differential Count

Normally, very few white blood cells are present in CSF (<5 cells/μL). A significant increase in white cells can be caused by infection. This analysis identifies and counts the different types of white cells that are present in a sample. Small numbers of lymphocytes and monocytes are normal in CSF. CSF leukocyte count is usually done manually with a hemocytometer counting chamber. A white cell count ≥5 cells/μL is considered elevated. Lymphocytes are most often elevated in cryptococcal meningitis; however, 30% to 50% of persons with cryptococcal meningitis will have an absence of CSF pleocytosis [22].

### CSF Red Blood Cell Counts

Normal CSF has no red blood cells. The presence of red blood cells in CSF may indicate bleeding in the central nervous system or a traumatic lumbar puncture.

### Lactic Acid

CSF lactate has been shown as a good indicator of severe meningitis [23]. CSF lactate levels will usually be increased with bacterial [24] and fungal meningitis, whereas they will remain normal or only slightly elevated with viral meningitis [25].

### India Ink Microscopy

**Figure 2.**
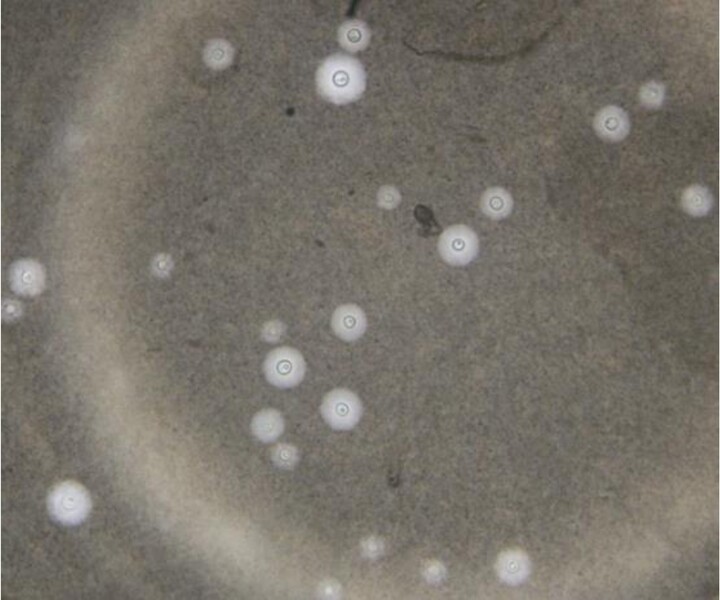
India ink staining. The background is stained, but the cells and capsule do not take up the stain.

We initially used CSF India ink staining for cryptococcal diagnosis. India ink uses the principle of negative staining. Under light microscopy, it stains the background but is not taken up by the yeast cells (Figure 2). Results can be obtained in 5 minutes. India ink is commonly used to demonstrate the presence of a capsule in *C neoformans*. India ink is used only on CSF and cannot differentiate between live and dead cells. India ink has not been used on other sample types, which limits its utility, especially when CSF cannot be obtained. This in turn limits early detection of disease, which contributes to the advancement of the disease. India ink has poor sensitivity at <90% [26].

Despite the limitations of India ink stain, it is cheaper for RCS to have it as a screening test, since most facilities have a light microscope, which may aid in patient care. India ink's shelf life and availability is within reach, hence making it a test that should be encouraged to be used in RCS.

### Other Microscopy Stains

CSF gram stain is performed as one of the basic microscopy procedures to demonstrate the presence of bacteria or fungi. In cryptococcal meningitis, budding yeast cells of *C neoformans* are seen, and the cells stain gram positive. Due to the poor diagnostic performance of India ink [26], other stains have been explored to improve on the sensitivity of detection for cryptococcal meningitis. Trypan blue is a stain used to distinguish between live and dead cells. Its use was explored in manual and automated platforms for the detection of cryptococcal cells in CSF. The TC20 automated cell counting (Bio-Rad) with trypan blue staining was poorly predictive of the quantitative CSF culture and could not be used as a substitute for quantitative culture [27]. Similarly, despite a positive correlation, the hemocytometer counts with trypan blue staining did not predict the outcome of quantitative cultures in patients receiving antifungal therapy [28]. However, acridine orange fluorescent microscopy was more sensitive than India ink light microscopy in the rapid detection of cryptococcosis among patients with CrAg-positive HIV [29].

### CrAg Latex Agglutination

The CrAg latex agglutination test is a qualitative and semiquantitative serologic test for the detection of capsular polysaccharide antigens of *C neoformans*. The latex agglutination test has diagnostic and prognostic value, since progressive disease is usually accompanied by increasing antigen titers [30]. The test can be performed on whole blood, serum, plasma, and CSF. It requires preheating of the sample, so it cannot be used in field settings or at the point of care. Results are obtained in 2 hours. However, it has a high rate of false positives [31].

### CrAg Lateral Flow Assay

**Figure 3.**
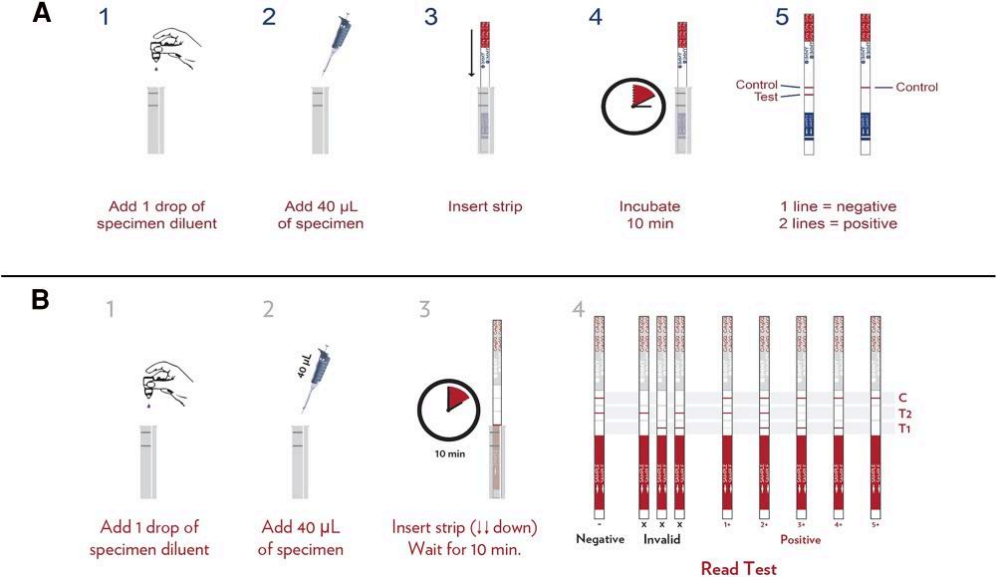
Procedures for cryptococcal antigen lateral flow and semiquantitative assays. *A*, Lateral flow assay takes 5 steps, and results are obtained in 10 minutes. *B*, Semiquantitative assay procedure. Images obtained from kit inset.

The CrAg lateral flow assay (LFA; Immy) is an immunochromatographic test system for the qualitative or semiquantitative detection of the capsular polysaccharide antigens of *Cryptococcus* species complex (*C neoformans* and *C gattii*) in serum, plasma, and CSF. The CrAg LFA can be used to conduct qualitative and semiquantitative tests [9, 32, 33]. Detection of CrAg in serum, plasma, and CSF has been extensively utilized with very high sensitivity and specificity [9, 34]. The CrAg LFA has excellent performance in CSF, serum, plasma [35], and fingerstick whole blood [7]. Other less invasive sample types have been explored with a demonstration of good performance in urine [36] but poor specificity in saliva [8]. The CrAg LFA is easy to use and meets the needs of small- and large-volume laboratories. It does not require laboratory infrastructure and can be stored easily at room temperature. No specimen pretreatment is required. The test also has the advantage of giving rapid results within 10 minutes. It is suitable for use in RCS (Figure 3*A*). The semiquantitative procedure for the CrAg LFA that gives antigen titers requires a laboratory setting and consumes more test strips with a longer turnaround time. There are other manufacturers of LFAs with suboptimal diagnostic performance [33, 37], which are not approved by the Food and Drug Administration.

### Semiquantitative CrAg LFA

The CrAg SQ (Immy) is a lateral flow semiquantitative assay that gives results in 10 minutes with a titer reading in a single test [33]. The CrAg SQ is a modification of the CrAg LFA, combining the qualitative and semiquantitative procedures into 1 kit. It is easy to interpret with training. It also has a positive correlation with CrAg titers determined by the CrAg LFA, colony-forming units (CFU) per milliliter from culture, and 10-week mortality [32] (Figure 3*B*).

### Enzyme Immunoassay

Enzyme immunoassay has been used to diagnose cryptococcal meningitis but mostly in research settings. It needs more sophisticated laboratory infrastructure (biosafety level 2 or 3). It has a turnaround time of 24 hours and a poor sensitivity [38, 39]. In RCS, samples may be batched to avoid wastage of reagents, which increases the turnaround time.

### Real-time Polymerase Chain Reaction

Real-time polymerase chain reaction (RT-PCR) has been a great advance in the diagnosis of cryptococcal meningitis. RT-PCR has high sensitivity and specificity, is fast, and can be done with small volumes of CSF [40]. Several RT-PCR platforms have been developed for the detection of *Cryptococcus* species [41, 42]. Some of them can distinguish between the 2 major species. Some are suitable for use in formalin-fixed, paraffin-embedded samples [43]. RT-PCRs have excellent performance with a turnaround time of approximately 4 hours. RT-PCR can be multiplexed to include agents of bacterial and viral meningitis. Examples include the BioFire FilmArray ME Panel (BioFire Diagnostics) [44].

### Histopathology

**Figure 4.**
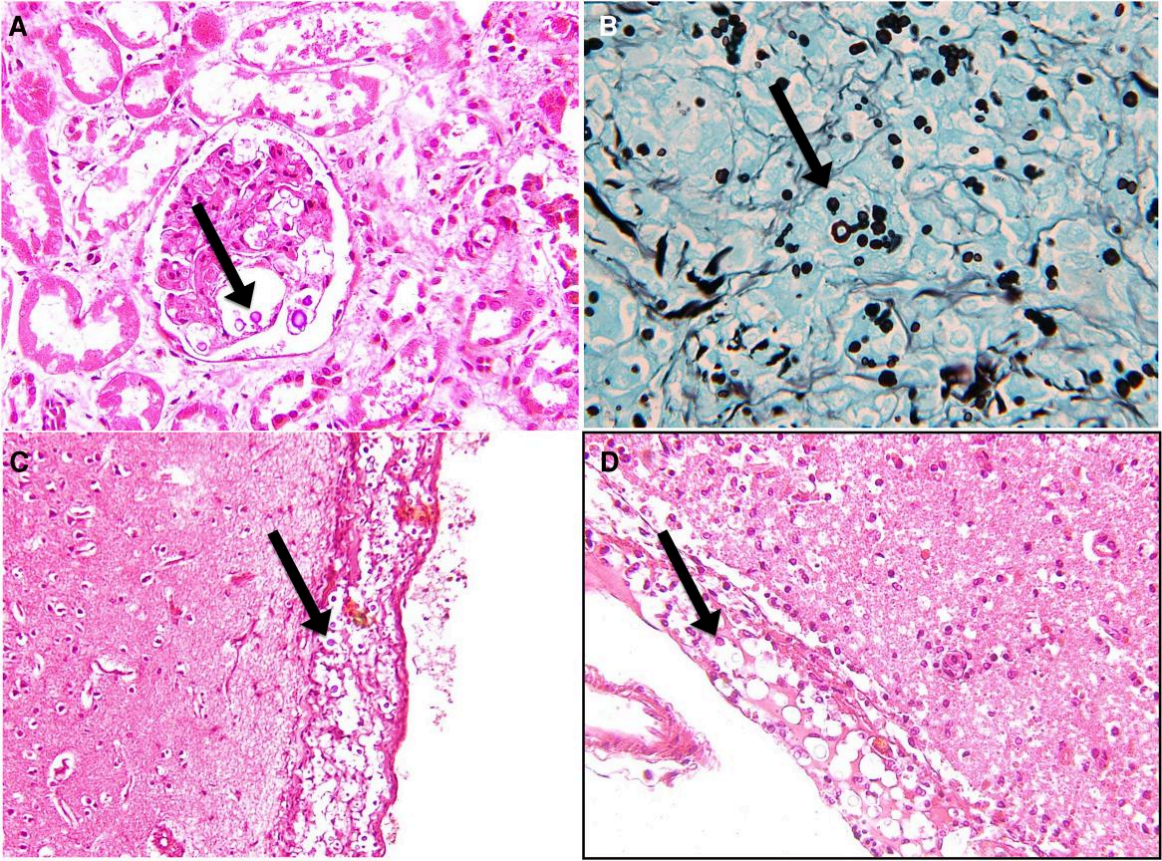
Histopathology sections. Section of the kidney showing *Cryptococcus neoformans* in the glomerulus: *A*, hematoxylin and eosin stain; *B*, Grocott methenamine silver stain. *C* and *D*, Section of the brain showing *C neoformans* in the meninges: hematoxylin and eosin stain. Black arrows indicate the location of the cryptococcal yeast cells in the tissue sections.

Histopathology is mostly used to diagnose disseminated disease with biopsies. It is also used in autopsy studies to establish the cause of death. Histopathology is based on the micromorphologic characteristics of *Cryptococcus* and includes histochemical techniques of hematoxylin and eosin and Grocott silver, as well as special histochemical stains [45] (Figure 4).

Table 1 shows a summary of the cryptococcal meningitis diagnostics, highlighting their diagnostic performances, availability in RCS, advantages, and disadvantages.

**Table 1.** Cryptococcal Meningitis Diagnostics

Test	Sample Type	Test Type	Diagnostic Performance	Availability in RCS	Advantages	Disadvantages
India ink	CSF	Qualitative	Poor sensitivity (50%–86%) Specificity (94%–100%)	Readily available in most RCS	Quick TAT Small sample volume Inexpensive	Cannot differentiate between dead and live cells Poor sensitivity Cannot differentiate between relapse and IRIS Uses CSF only
Trypan blue	CSF	Qualitative	Poor sensitivity (95%–98%) Specificity (unknown)	Rare; mostly available in research settings	Quick TAT Small sample volume Inexpensive	Poor sensitivity Limited sample types; uses CSF only
Acridine orange	CSF	Qualitative	Good sensitivity (96%) Specificity (unknown)	Rare	Quick TAT Small sample volume Good sensitivity	Expensive
Latex agglutination	WB, serum, plasma, and CSF	Qualitative and semiquantitative	Good sensitivity (87%–100%) Specificity (68%–98%)	Occasional	Good performance	Cannot be used outside a laboratory Need to preheat the sample Gives false positives Long TAT
Culture	CSF	Quantitative	Excellent sensitivity (85%–94%) Specificity (unknown)	Common	Inexpensive Gold standard	Long TAT May give false negatives with low antigen concentrations
Enzyme immunoassay	CSF	Quantitative	Poor sensitivity (55%–100%) Specificity (60%–100%)	Rare; mostly available in research settings	Small sample volume	Expensive
Lateral flow assay	CSF, serum, plasma, WB	Qualitative and semiquantitative	Excellent sensitivity (100%) Specificity (100%)	Common	Quick TAT Small sample volume Inexpensive Used in field conditions	False negatives due to hook effect (prozone) Semiquantitative procedure requires laboratory setting and consumes more test strips with a longer TAT Cannot differentiate between CM-IRIS and relapse
SQ LFA	CSF, serum, plasma, WB	Qualitative and semiquantitative	Excellent sensitivity (100%) Specificity (100%)	Not yet rolled to lower health centers	Quick TAT Small sample volume Used in field conditions	Cannot differentiate between CM-IRIS and relapse
PCR	CSF	Quantitative	Good sensitivity (50%–100%) Specificity (100%)	Rare	Small sample volume	Cannot be used outside a laboratory Long TAT
Histopathology	Tissue	Qualitative	Poor sensitivity (unknown) Specificity (unknown)	Common; mostly used in autopsy cases	Demonstrates tissue invasion	Long TAT Not used in routine diagnosis Limited to tissue samples
API	Isolates	Qualitative	Excellent sensitivity (90%–100%) Specificity (unknown)	Rare	Standardized Longer shelf life	Needs ≥48 h Subjective Human error

Abbreviations: API, Analytical Profile Index; CM, cryptococcal meningitis; CSF, cerebrospinal fluid; IRIS, immune reconstitution inflammatory syndrome; LFA, lateral flow assay; PCR, polymerase chain reaction; RCS, resource-constrained settings; SQ, semiquantitative; TAT, turnaround time; WB, whole blood.

## OPTIMIZING CRYPTOCOCCAL MENINGITIS TREATMENT

### Role of Early Fungicidal Activity

The antifungal activity of a regimen can be quantified by the rate of CSF fungal clearance by serial quantitative CSF fungal cultures. This early fungicidal activity is quantified as the change in log_10_ CFU per milliliter of CSF per day of therapy. In general, a slower rate of fungal clearance is linked to increased mortality, particularly when the early fungicidal activity is worse than 0.20 log_10_ CFU/mL per day [46, 47].

### Role of CrAg Titers

Previous studies have shown that approximately 6% of patients with HIV and a CD4 count ≤100 cell/μL have cryptococcal antigenemia and so may need titers [48]. About 7% of patients will have symptomatic cryptococcal antigenemia presenting as early cryptococcal meningitis with 38% mortality [49]. Patients with CrAg titers ≥1:160 had poor treatment outcomes, and the majority progressed to cryptococcal meningitis. Patients with titers <1:160 had good treatment outcomes. Patients with high titers require high-dose fluconazole and a lumbar puncture to rule out central nervous system disease [50]. Data on the value of titers in monitoring treatment are limited.

### Role of Antifungal Susceptibility Testing

Research into mechanisms of antifungal resistance for *C neoformans* is limited. The molecular basis of resistance to azoles remains poorly understood. Fluconazole was previously considered the most effective maintenance therapy for cryptococcal meningitis. However, treating cryptococcosis has become more challenging in recent years due to triazole resistance [51]. Studies from Uganda showed 31% of *C neoformans* clinical isolates having a fluconazole minimum inhibitory concentration (MIC) ≥16 μg/mL. Fluconazole MIC values tended to increase over the course of treatment, but a correlation with clinical failure has not been established [52]. Many recent studies suggest an increase in triazole resistance by *C neoformans*, especially to fluconazole, which is widely used in the management of cryptococcal meningitis [53].

Knowing local antifungal susceptibility patterns can inform the attending physician when making therapeutic choices. Antifungal prescribing may need individualization for each patient diagnosed with cryptococcal meningitis. Data from antifungal susceptibility testing can be used epidemiologically at a national level for surveillance to determine the susceptibility profiles for local fungal pathogens, which can in turn guide and inform antifungal stewardship programs. These data can also be used to define local cutoff values.

Methods for antifungal susceptibility testing have evolved greatly from the traditional broth dilution and disc diffusion to gradient strips (e-test), agar-based screening, Vitek 2 system, colorimetric broth microdilution (Sensitire YeastOne), Micronaut AM, ATB Fungus 3, PCR, flow cytometry, matrix-assisted laser desorption ionization–time of flight mass spectrometry, porous aluminium oxide–based culture, and isothermal microcalorimetry [54].

### Role of Therapeutic Drug Monitoring

Therapeutic drug monitoring is mostly indicated in patients whose therapy is failing, despite receiving the optimal standard of care. Treatment failure is more common in patients receiving fluconazole monotherapy [55]. Therapeutic drug monitoring can be done after ruling out antifungal resistance. A population pharmacokinetics model is the first critical step for revising antifungal drug regimens that maximize fungal killing and minimize toxicity and the emergence of antifungal resistance. The pharmacokinetics of antifungal drugs, especially fluconazole, may vary from one patient to another [56]. It may be helpful to adjust the doses and customize them for each patient to optimize treatment outcomes. Caution must be taken to minimize toxicity.

MICs are a better indicator to guide the appropriate use of fluconazole [57]. However, therapeutic drug monitoring and antifungal susceptibility testing are not done routinely in RCS. They are occasionally done under research settings in RCS. Flucytosine pharmacokinetics levels also show significant interindividual variability in plasma and CSF among patients with HIV-associated cryptococcal meningitis [58]. Liposomal amphotericin B concentrations are highly variable with no evidence of nonlinear pharmacokinetics at a dose of 10 mg/kg [59]. Therefore, therapeutic drug monitoring helps to monitor and optimize the dosing of antifungals for cryptococcal meningitis treatment to optimize treatment outcomes.

## MISSING LINKS TO OPTIMIZE DIAGNOSIS AND OUTCOMES IN RCS AND PRACTICAL RECOMMENDATIONS

With the continuous evolution of laboratory diagnostics for cryptococcal infection, we noticed a few gaps that need to be addressed to improve cryptococcal disease diagnosis and outcomes.

CSF fungal culture is the historical gold standard, yet it has a long turnaround time of 7 to 14 days. This delays diagnosis and treatment in RCS that have culture as the only test available. Most RCS with culture facilities do not differentiate between the 2 species of cryptococcus. Besides, sensitivity for fungal culture is 90%, and there are other point-of-care tests with better sensitivity of up to 100%. Therefore, we need to find an alternative gold standard for cryptococcal disease with an excellent diagnostic performance and short turnaround time.

Currently, fungal culture is the only test available for distinguishing between cryptococcal meningitis relapse and cryptococcal meningitis IRIS. Yet, culture has a long turnaround time. This delays clinical decision making, resulting in longer hospitalization and subsequently increasing the risk of nosocomial infections and thus poor outcomes. We need an alternative test to quickly distinguish between cryptococcal meningitis relapse and cryptococcal meningitis IRIS.

Some RCS—especially in sub-Saharan Africa, where the disease burden is highest—still have no access to the point-of-care CrAg LFA. This test needs to be added to the essential diagnostics list of each country to be provided by HIV programs, especially those with a high burden of HIV. Since the cost of the tests is usually a major limitation to access, there is a need to engage manufacturing companies to provide it at a subsidized price for RCS, where the burden of disease is higher and so provides a good market base for these companies.

Delayed diagnosis for cryptococcal infection is still a major problem in RCS, with >90% of patients presenting late for diagnosis. This is partly due to a lack of access to proper diagnostics and the low index of clinical suspicion. There are many missed opportunities that require boosting screening programs. Most patients who are symptomatic spend 1 to 4 weeks in clinics or self-medicating with painkillers, antibiotics, and antimalarials before referral to the hospital, where they are then diagnosed with HIV-associated cryptococcal meningitis. In most RCS, the limited availability and access to fungal diagnostics is probably due to a lack of laboratory facilities, a lack of trained personnel to perform the tests, the high cost of tests, a lack of antimicrobial stewardship, and a perceived lack of need among policy makers. CrAg titers are a semiquantitative way of quickly measuring the fungal burden in clinical samples. However, their role in monitoring treatment needs further investigation. It has been suggested that serum CrAg titers could be used to predict meningitis in RCS where lumbar puncture cannot be performed; the feasibility of this approach should also be further investigated in a diversity of settings. This could provide an alternative to quantitative cultures in estimating fungal burden.

In RCS, very few health facilities at the regional or national referral level have the capacity to perform lumbar punctures to obtain CSF for diagnostic microbiology and reducing intracranial pressures. A related controversy is whether we can obtain a less invasive sample than CSF that can provide similar diagnostic value. The refusal rate for lumbar punctures is still a concern in many RCS. There is a need to train more health workers at all levels to perform lumbar punctures or improve the referral system to higher facilities that can perform the procedure.

*Cryptococcus* species have intrinsic resistance to echinocandins [60]. Studies in Uganda showed that 31% of *C neoformans* clinical isolates had fluconazole MICs ≥16 μg/mL [52]. However, in most RCS, antifungal susceptibility testing is generally not available for routine antifungal resistance testing. A recent survey on the current state of clinical mycology showed that only 30% and 39% of institutions have access to antifungal susceptibility testing in Africa [61] and Latin America and the Caribbean [62], respectively. A similar survey done in Asia/Pacific showed that only 48.5% of the countries have the Clinical and Laboratory Standards Institute available, 15.7% have EUCAST, 37% have e-test, and 49.8% have Vitek [63]. Besides, AFST is costly and cannot be afforded by most RCS. It also relies on having a positive culture in most circumstances. Access to quality control strains is an additional challenge in RCS. So, there is a need to improve access to antifungal susceptibility testing in RCS. A test that simultaneously detects CrAg and fluconazole resistance would be a great addition.

Related to the aforementioned, therapeutic drug monitoring is not available in RCS to check for antifungal drug levels, which would help to inform on the appropriate dosing for each patient to avoid toxicity, resistance, and poor treatment outcomes.

With the variety of comorbidities and opportunistic infections usually seen in patients with HIV-associated cryptococcal meningitis, there is no test that can simultaneously detect cryptococcosis and related opportunistic infections, such as tuberculosis, histoplasmosis, aspergillosis, pneumocystis pneumonia, toxoplasmosis, and Kaposi sarcoma. Such a test would be very helpful in advanced HIV disease.

Other challenges that are likely to cause delayed diagnosis in RCS include the use of alternative medical practices, stigma, economic challenges, and frequent transfer of the trained laboratory staff. Other salient factors that contribute to challenges in diagnosis include the patient's environmental exposure (eg, birds), indoor environment, and occupation leading to recurrent infections. Poor equipment maintenance, procurement challenges, and competing national budget allocations also contribute to diagnostic challenges.

## CONCLUSIONS

Diagnostics for cryptococcosis have evolved over time from complex laboratory-based tests to simple tests that can be used at the point of care. Early diagnosis of cryptococcal meningitis remains crucial in improving treatment outcomes. Fungal culture of CSF is the historical gold standard for the diagnosis of cryptococcal meningitis but has a few limitations. Microscopy is limited by poor sensitivity. Detection of CrAg has improved drastically with the point-of-care CrAg LFA and CrAg SQ. Other cryptococcal meningitis diagnostics are mostly limited by the high cost and limited access in RCS. Early fungicidal activity and CrAg titers could be alternative ways of quantifying fungal burden. Antifungal susceptibility testing and therapeutic drug monitoring are essential for optimizing treatment, but they have limited access in RCS.

The major missing links to optimize treatment outcomes among patients with cryptococcal meningitis involve troubleshooting the limitations of fungal culture, addressing delayed diagnosis, and developing more versatile diagnostics, as well as improving access to the CrAg LFA, antifungal susceptibility testing, and therapeutic drug monitoring. However, from RCS we find that most facilities have a light microscope, since it is still an essential tool in diagnosis. Improved or trained human resource capacity in the identification of cryptococcal meningitis is still key. Procurement issues too affect the availability of tests, unless RCS are funded by outside sources. There remain issues of inequality and equity of resource allocation between private and government-run laboratories. The high cost of tests is still a limiting factor to access in RCS. Implementation of these tests in RCS is also hindered by competing diseases and the health systems of these settings, which in turn affect diagnosis of cryptococcal meningitis.

## Notes

***Acknowledgments.*** We are grateful for institutional support from the Makerere University Infectious Diseases Institute. We acknowledge the review and input from David Boulware.

***Author contributions.*** R. K. conceived the study and designed the concept. R. K. and T. K. K. collected data. R. K. and T. K. K. participated in the initial manuscript drafting. All authors participated in critical revisions for intellectual content. D. B. M. participated in project administration and supervision. All authors approved the final version of the manuscript.

***Availability of data and materials.*** The authors confirm that all data underlying the findings are included in the article and its supporting documents.

***Financial support*.** This work was supported by the National Institutes of Neurological Disorders and Stroke; and the Fogarty International Center (R01NS086312, R01NS110519, D43TW009345).
